# Supplementary material for: Are carbon emissions trading and green financial instruments synergistic? -Comprehensive quantitative research based on content analysis
Source: PLoS One. 2024 Mar 7;19(3):e0298601. doi: 10.1371/journal.pone.0298601 (PMC10919601; doi:10.1371/journal.pone.0298601)
Supplement: S1 Data — (ZIP) [file pone.0298601.s001.zip › Data/地级市Z_F工作报告.zip!/╣π╢1⁄2/╟σ╘╢╩╨/└┤╫╘╣1⁄2╓┌║┼í╛┬φ┐╦╩2╛▌═°í┐_2008.docx]

各位代表：

我代表市人民政府向大会作政府工作报告，请予审议，并请政协各位委员和其他列席人员提出意见。

2007年工作回顾

2007年，市政府在省委、省政府和市委的正确领导下，在市人民代表大会及其常务委员会和市政协的监督支持下，高举中国特色社会主义伟大旗帜，坚持以科学发展观统领经济社会发展全局，深入贯彻落实党的十六届六中、七中全会、十七大精神，紧紧围绕争当山区发展排头兵目标，迎难而上，开拓创新，扎实工作，克服各种困难，经济社会实现又好又快发展，取得了主要经济指标增幅全省“四连冠”、六项经济指标总量分别居山区市第一的喜人成绩。

一、经济快速增长，发展质量显著提高

经济实力再上新台阶。全市实现地区生产总值（GDP）597亿元，比上年增长32.3%，增幅居全省第一；总量继续居山区市首位，列全省第13位；人均GDP1.64万元，增长31%。工业经济大幅增长，全社会工业总产值1176亿元，其中规模工业总产值进入全省前十位，达1108亿元，增长79.7%；规模工业增加值277亿元，增长59.9%，增幅居全省第一。第三产业完成增加值184.6亿元，增长21.4%，增幅创八年来新高。其中，全年接待游客1323万人次，增长19.9%；旅游收入47.9亿元，增长33.4%。房地产业完成投资64.9亿元，增长65.2%；全年房屋销售面积239.7万平方米，增长71.2%。城乡市场兴旺，实现社会消费品零售总额197亿元，增长23.5%，增幅创十二年来新高，居全省第一，消费规模跃居山区市首位。

县镇两级经济发展实现新突破。各县（市、区）生产总值增幅均超过全省平均水平。市辖区人均生产总值达3.4万元，率先赶超全省平均水平。英德市、清新县生产总值均突破百亿元大关。佛冈县人均生产总值突破2万元，进入全省67个县（市）前十名。有四个县（市、区）工业总产值突破百亿元大关，其中清城区突破400亿元，佛冈、清新两县突破200亿元，英德市突破100亿元。镇级经济快速发展，有14个镇街工业总产值突破20亿元，其中清城区龙塘镇突破200亿元，清城区石角镇、佛冈县石角镇以及清新县太和镇突破100亿元。工业十强镇完成工业总产值854亿元，占全市工业七成以上。

基础设施建设力度加大。全年完成固定资产投资487亿元，增长48%，按省统计口径计算，增幅居全省第一；投资规模位列全省第六。其中，交通邮电通信完成投资71亿元，增长28.7%。武广铁路客运专线、第二个“八路一桥”等项目取得重大进展。北江大桥扩建工程建成通车；清连公路高速化改造已完成单幅路面施工，今年底可望全线建成通车；石龙和德龙产业大道、国道323线连山吉田至鹰扬关段改造、省道261线连南三江至水足塘段改建等工程进展顺利。清远城西大道已动工建设，广清高速公路四车道改八车道扩建工程工可通过专家评审，广乐高速公路建设提前列入省“十一五”规划加快实施，二广高速公路经连山、连南县城线路方案已获通过。水利设施建设加快，全年共完成投资5.82亿元，省人大议案174宗小型水库除险加固任务全面完成，实现了五年任务三年完成目标；全市20宗城乡水利防灾减灾工程项目已开工建设19宗，农村机电排灌工程和农村饮水安全工程应急项目建设顺利推进。基础产业完成投资196亿元，增长63.3%。133个重点建设项目中，已竣工投产或部分投产22个，在建94个。清远抽水蓄能电站、阳山燃煤发电厂等项目前期工作取得重大突破。城乡建设日新月异，各县（市）城镇配套设施不断完善，美化、绿化、亮化水平不断提高；以“五个一批”为重点的市区基础设施建设步伐加快，其中，中山公园改造工程基本完成；建成精品住宅小区一批；“一江两岸”城市园林景观进一步完善；城市排污、生活垃圾处理能力显著增强，市容环境和居住环境明显改善。

产业结构调整力度加大。三次产业增加值比例由上年的18:48:34调整为15:54:31，第二产业比重上升了6个百分点。农业结构进一步改善，粮经比例为30:70，种养比例为60:40。

经济效益大幅提升。规模工业企业亏损面由上年的45%下降到39%，实现利润总额21.5亿元，增长57%；工业经济效益综合指数191.6，提高32.3个百分点，提升幅度居全省第二。财税收入大幅增加，来源于清远的财政总收入达63.2亿元，增长42%；国税、地税共组织税收收入55.4亿元，增收14亿元；地方一般预算收入27.9亿元，增长42.3%，增幅居山区市第一、全省第二；金融运作积极稳健，年末金融机构各项存款余额559.9亿元，比年初增长18.9%；金融机构贷款余额235.5亿元，增长23.9%。

品牌创建步伐加快。相继荣获了“港澳及海外华人眼中最具魅力的休闲度假之都”、“中国生态休闲旅游城市”和“中国宜居城市”等称号。清远温泉和漂流被评为“欧洲人最喜爱的中国景区”，清新县被授予“全国旅游强县”称号，阳山县和连州市获“广东省旅游强县”称号，新创建国家“4A”级景区4个。生产基地和产品类品牌再创佳绩，被授予“中国再生铜都”称号。新增“国家免检产品”2个、“广东省名牌产品”12个、“广东省著名商标”6件、绿色食品A级认证产品2个、有机食品认证1个、无公害农产品产地认定122家、认证无公害农产品171个、国家地理标志产品5个。

环境保护和节能降耗成效明显。全市财政用于环保支出5601万元，增长59.8%。大力推行清洁生产，深入开展整治违法排污企业、保障群众健康环保专项行动，关停二氧化硫和化学需氧量重点污染企业20家。严把项目环保审批准入关，禁止建设不符合规划和产业政策的项目；实施燃煤电厂脱硫、垃圾无害化处理等环保重点工程；积极推进污水处理厂建设，旧城区（清新）、连州市和佛冈县污水处理厂投入试运行；开展矿产资源开发整治，关闭违法采矿区129个，复绿60个。“市肺”工程进展顺利，总体规划已通过评审；完成迹地更新造林24.5万亩，全市森林覆盖率达68%。全市空气质量良好，乐排河、大燕河水质明显改善，主要江河水质基本保持国家Ⅱ类水质标准，各县（市、区）饮用水源水质达标率在99%以上。大力推广节能减排新工艺、新技术，积极发展循环经济。狠抓节能降耗目标责任制落实，积极推进重点行业和重点企业节能工作，对全市首批100家重点耗能企业实行重点监控，与列入全省千家节能行动的49家重点耗能企业签订节能目标责任书。全市单位生产总值能耗下降3.6%，其中工业增加值能耗下降26.7%。

二、农业农村经济平稳发展，新农村建设扎实推进

完成农林牧渔业产值136亿元，比上年增长6.4%。成功举办了第三届中国华南（清远·清城）农业博览会。

综合生产能力进一步增强。加快现代标准农田建设，从2007年起，每年建设现代标准农田12.5万亩。大力推进农业机械化，全市农机总动力达到91.17万千瓦，增长2.5%。加快推进农业标准化生产，全市已建立2个国家级农业标准化示范区、13个省级农业标准化示范区，正在立项制订地方标准4个。农业产业化扎实推进，全市具有一定规模的农业产业化经营组织149家，实现年销售收入36亿元，出口创汇3092万美元，带动27.4万农户增收7.43亿元。

社会主义新农村建设取得新进展。坚持市、县（市、区）、镇三级示范的原则，积极开展“反哺农村”行动，“八个五”工程建设成果进一步扩大，80个市级社会主义新农村示范村建设初见成效，涌现出一批新农村建设先进典型。

三、改革开放力度进一步加大，内外源型经济同步发展

各项改革积极稳妥推进。着力抓好行政审批制度改革，行政审批电子监察系统投入运行，全市共清理行政审批事项2973项，取消1052项。城信社退市工作稳步推进。农信社票据兑付工作进展顺利，连州、连山、连南、阳山、清新和佛冈等六家联社已全部完成票据兑付。积极推进国有企业资产保值增值和特困国有企业改革，基本完成了粮食流通体制和国有粮食企业改革。积极推进综合行政执法和乡镇国土所管理体制改革。深化财政管理体制改革，镇财县管工作顺利开展。

民营经济迅速发展。全市新增个体工商经营户2.28万户，新增注册资金4.01亿元；新登记私营企业1168家，新增注册资金18.2亿元。民营经济全年完成投资225亿元，增长45.4%，占全市固定资产投资总额的46%。规模以上民营工业企业完成增加值112亿元，增长81%；实现税金总额5.05亿元，增长69%。

招商引资成效显著。全年共引进项目671个，合同投资金额544亿元。目前已投产项目144个，在建项目229个，实际投入资金252亿元。新批外商投资项目109个，实际利用外资4.46亿美元，增长37.3%。江西铜业、云南铜业等一批带动能力强、科技含量高、财税贡献大的工业项目相继落户我市。产业转移园区建设取得实质性进展，佛山（清远）产业转移园区已被省政府认定。各县（市、区）也与珠三角地区签订了联合共建产业转移园区合同。

四、深入推进大文化建设，社会各项事业全面进步

教育、文化、体育事业快速发展。全市财政用于教育事业支出15亿元，增长18.2%。“两基”工作进一步巩固，272所学校通过规范化学校初评；高中扩容促优成效显著，高中阶段毛入学率达到56%；农村学校危房改造全面完成，并顺利通过省验收。教育创强工作成绩显著，办学质量明显提高。清城区和佛冈县创建省级教育强区、强县工作进展顺利。全市现有省教育强镇5个，名列全省第8位，居东西两翼和粤北地区首位。全市有8所国家级示范性高中，省、市一级学校98所，中小学优质学位18.38万个。职业教育快速发展。文化事业日益繁荣，广场文化艺术节已成为清远品牌；广播电视和新闻出版事业上新水平。瑶族长鼓舞、英石假山盆景传统工艺被列入“中国非物质文化遗产”，“狮子舞”等7项民俗特色文化项目被列入“广东省非物质文化遗产”。启动了创建全国文明城市工作。积极开展“全民健身与奥运同行”活动，精心组织了以“绿色清远·活力无限”为主题的18项大型文体系列活动。

卫生、科技、计划生育事业加快发展。全市财政用于科技、医疗卫生事业的支出各为3062万元和4.4亿元，分别增长13%和76%。医疗救治体系建设步伐加快，应对突发公共卫生事件能力进一步提高。公共卫生体系建设全面加强，疾病预防控制和医疗救治项目基本完成，妇幼卫生和爱国卫生深入开展；农村和社区卫生稳步发展；医疗卫生条件明显改善，市疾控中心等一批卫生建设项目建成投入使用，市人民医院新院区建设进展顺利，农村卫生院“改薄”工作有效推进。自主创新能力不断增强，建成我市首家省级工程研发中心“省电力线缆工程技术研究开发中心”和首家“省农业科技创新中心”。新增省高新技术企业4家、省民营科技企业4家；7家企业12个产品被评为省高新技术产品；新批准2个省级技术创新专业镇。信息化步伐不断加快，电子政务建设取得新进展。计划生育层级动态管理责任制进一步落实，多层次计划生育利益导向机制进一步完善，人口出生率为11.47‰，自然增长率为6.02‰，完成省责任制目标任务。

各项社会事业全面进步。加强国防建设和民兵预备役工作，认真做好双拥工作，落实优抚安置政策，军地合作共建进一步加强。统计、外事、侨务、对台、人事、妇女儿童、红十字、扶贫、人防、气象、地震、档案、方志、殡改等各项工作得到加强。

五、着力改善民生，人民群众享受更多发展成果

2007年，全市财政用于民生方面的支出达33.86亿元，占全年财政支出的54.65%。

解决农村“一保五难”问题成效显著。农村人均收入低于1200元的困难家庭全部列入低保救济；完成2007年省下达给我市的2150户农村危房改造任务；全市农村新型合作医疗参合率达到97%；镇通村公路完成938公里硬化建设；全市小学入学率99.9%，初中入学率97.7%；完成农村饮水工程104宗，解决5.95万群众的饮水困难问题，受益人数达13.72万人。

惠民政策得到落实。全市农林水事务支出6.43亿元，增长25.5%。其中，种粮等五种补贴8023.96万元。全市财政共支出2638万元，免除农村在校学生和城市特困家庭子女义务教育学杂费。从2007年秋季起，对就读全日制学历教育的中职一、二年级农村学生和城市困难家庭子女，每人每年补助1500元生活费。

就业工作成效明显。认真抓好技能培训工程，全市累计培训富余劳动力3.86万人次，培训退役士兵768名，劳动者技能水平、就业能力明显提高。全市新增就业岗位8.23万个，转移农村富余劳动力13.44万人次，零就业家庭首次实现动态为零，城镇登记失业率下降到2.71%。

社会保障体系进一步完善。城镇各项社保改革不断深化，制度逐步完善。国企下岗职工基本生活得到妥善安置，离退休人员基本养老金按时足额发放并逐年提高，全市累计参加养老保险34.8万人、失业保险14万人、医疗保险22.2万人、工伤保险23.3万人、生育保险9.9万人，分别增长15.05%、5.88%、13.45%、21.05%、10.42%。全年共征缴各项社会保险费10.29亿元，增长25.52%；支付离退休人员各项社保费用7.22亿元，增长24.92%。各项社保基金累计结余16.63亿元，增长22.4%。切实保障劳动者合法权益，解决拖欠农民工工资985.45万元。城乡低保实现全覆盖，全市有14.04万困难群众纳入低保救济。建立家庭经济困难大学新生助学机制，慈善事业得到社会各界的关心和支持。

人民生活明显改善。全市在岗职工平均工资2.36万元，增长16.5%。农民人均纯收入4281元，增长9.2%。城乡居民储蓄存款余额367.8亿元，比上年末增长12.4%。

六、和谐清远建设步伐加快，社会保持稳定

民主法制建设进一步加强。深入开展“五五”普法教育，加强法律援助，全民法律意识和法制观念有新提高。完善村民自治制度，坚持政务公开、厂务公开、村（居）务公开，人民群众参与民主管理、行使民主监督的权利得到保障。全面推进依法行政，政府法制工作取得新成效。自觉接受市人民代表大会及其常务委员会的法律监督、工作监督和市政协的民主监督，坚持重大事项向市人大常委会报告、向市政协通报，注重发挥各民主党派、工商联、人民团体和无党派人士的参政议政作用，共办复人大代表建议85件、政协提案183件、政协委员来信38件，办复率达100%。贯彻落实党的民族、宗教政策，用办特区的理念加快少数民族地区发展取得明显成效。

社会管理成效显著。土地利用规划进一步完善，土地供求矛盾有所缓和。完善各项制度，政府采购、预算资金使用、工程招投标、土地招拍挂等行为更加规范。加大审计监督力度，财政资金使用效益进一步提高。严格控制政府提价措施出台，居民消费价格指数低于全国、全省平均水平。产品质量和食品安全专项整治工作取得显著成效，完成了12个100%的目标任务，涉及人身安全与健康的食品、药品等监管取得突破性进展。落实安全生产监管责任，开展事故隐患排查整治行动，全市各类事故起数、死亡人数、受伤人数同比分别下降18.5%、16.7%、21.7%，没有发生重特大事故。落实信访工作责任制，着力抓好“21项民生信访问题专项治理”，重点信访难题得到解决，越级上访明显减少。打击传销工作成效显著，创建无传销县（市、区）4个，无传销镇（街道）80个。强化社会治安防控工作，加强社会治安综合治理，开展命案侦破、打黑除恶、打击“两抢两盗”等专项行动，维护了社会稳定。

各位代表，过去一年，发展的历程令人难忘，发展的成就令人振奋。这是全市人民在市委正确领导下团结奋斗的结果，是市人大、市政协加强监督、鼎力支持的结果，是港澳台同胞、海外侨胞和社会各界关心支持的结果，是兄弟地区尤其是对口帮扶地区热心支持的结果。在此，我代表市人民政府向全市人民、人大代表、政协委员，向各民主党派、工商联和无党派人士、人民团体，向驻清解放军和武警官兵、国家和省驻清单位，向海外侨胞、港澳台同胞以及所有参与、支持和关心清远建设的海内外朋友，表示衷心的感谢，并致以崇高的敬意！

我们也清醒地看到，当前我市经济社会发展面临不少困难和问题：一是我们的思想观念、体制机制还不适应科学发展的要求，需要进一步解放思想，建立符合科学发展要求的体制机制；二是区域发展不协调仍然突出，北部地区受交通等因素制约，自我发展能力较低，需要加大扶持和带动力度；三是产业结构还不合理，自主创新总体能力不强，节能减排压力较大，转变经济增长方式的任务艰巨；四是基础设施还不能满足经济社会发展的需要，资金、土地、能源供应紧张仍是制约发展的瓶颈；五是部分困难群众生活压力较大，需要进一步改善民生、纾解民困等等，我们必须高度重视解决这些问题。

2008年工作安排

2008年，是改革开放30周年，也是清远建市20周年。如何在快速发展的基础上，向更高发展目标迈进，我们面临的机遇和挑战并存。一方面，我们要清醒认识继续加快发展的困难因素。全球经济波动，将对我们的外向型经济造成一定影响。同时，国家加大宏观调控的力度没有松动，继续实行从紧的货币、土地供应政策，严格控制新开工项目等，我们在发展中将会遇到一些预想不到的问题。此外，年初的低温冰雪灾害，给我们造成了巨大损失，将增加我们发展的难度。另一方面，我们更要看到发展的有利因素。今年，国家和省将进一步加大区域协调发展力度，大幅度增加对社会保障和救济、卫生、教育等方面的支出；对欠发达地区实施一系列积极的扶持政策，继续加大对欠发达地区基础设施建设的支持力度、财政转移支付力度、产业转移力度等，将给我们提供发展的动力。另外，经过20年的建设和发展，我市经济社会发展的基础日渐厚实，自我发展的能力显著增强。同时，我们所具有的资源优势、区位优势和政策优势还没有得到充分利用，发展的潜力还很大。因此，我们既要认真研究分析国家宏观调控对我市经济社会发展的影响，更要灵活运用好各种支持性政策；既要居安思危，深谋远虑，更要增强信心，加快发展。

今年工作的总体要求是：全面贯彻落实党的十七大精神，按照省委十届二次全会和市委五届五次全会的部署，围绕经济建设这个中心，突出科学发展与和谐社会两大主题，以解放思想、改革开放为动力，提高推动科学发展能力；以调整经济结构为重点，提高经济发展质量；以加大建设力度为手段，提高基础设施保障能力；以完善规划为引领，提高城市建设管理水平；以新农村建设为载体，提高城乡协调发展水平；以改善民生为追求，提高群众生活水平，不断开创经济社会又好又快发展的新局面，努力争当山区科学发展排头兵。

今年全市经济社会发展的主要预期目标是：地区生产总值增长20%左右；工业增加值增长28%；单位生产总值能耗降低3.5%；二氧化硫和化学需氧量分别完成省定减排目标；地方一般预算收入增长25%；全社会固定资产投资增长25%；实际利用外资增长25%；外贸出口增长10%；社会消费品零售总额增长18%；居民消费品价格涨幅控制在4%以内；城镇登记失业率控制在3.5%以内；在岗职工平均工资增长12%；农村居民人均纯收入增长9%；人口自然增长率控制在6.8‰以内。

具体抓好以下十项工作：

一、加大区域协调发展力度，提高经济发展水平

切实做好灾后复产重建工作。当前，我们已全面进入复产重建的重要阶段，各地各部门要继续做到思想不松劲、力量不减弱，切实加强对复产重建工作的组织领导，做好服务，确保复产重建有序进行，力争大灾之年不减产、不减收，把损失降至最低。要及时组织和动员科技人员深入灾区一线，进村入户开展技术培训，指导农民开展补种、改种农作物，指导林区开展清理、补种补造。要因地制宜，结合新农村建设制定重建家园方案，加快建设，确保全倒户六月底前搬进新居。

加大扶贫开发力度。认真落实好汪洋书记考察清远时关于扶贫工作的讲话精神，创新扶贫方式，按照“规划到户、责任到人”的要求，对全市农村贫困户实行登记造册、电脑管理，深入开展“一户、一策、一干部”帮扶活动。同时，要把帮扶活动与指导贫困农民就业、创业有机结合起来，增强贫困农民自我发展能力，共建经济发展成果。

加快发展县域经济。因地制宜，分类指导，促进县域经济加快发展。加快南部地区产业升级步伐，大力发展新型制造业，积极培育高新技术产业，不断壮大高新技术产业集群。加大对北部山区和少数民族地区基础设施建设扶持力度。北部山区要充分利用清连高速公路通车在即的机遇，掀起新一轮发展热潮，把资源优势转变为产业优势，把产业优势转变为竞争优势，加大下游产业发展力度，进一步提高资源综合利用效益，形成特色经济。连州、英德两个次中心城市要以城市建设为引领，以招商引资为重点，以培育市场要素为带动，加快改变产业单一现状，提高经济抗风险能力，增强财富积聚功能和辐射带动能力。

二、加大结构调整力度，提升经济发展质量

大力发展民营经济。全面贯彻落实扶持民营经济发展的政策措施。继续实施中小企业成长工程。推动政银企合作，建立多层次的融资担保体系，积极开展面向中小企业的融资和小额信贷。引导民资广泛参与产业发展、基础设施、公用事业和社会事业建设，鼓励民营企业加快制度、技术、管理和品牌创新，支持骨干民营企业做大做强，提高产业层次和水平。

优化对外贸易结构。完善重点出口企业工作联系制度，及时掌握企业生产和出口信息，提高口岸通关效率，提升对外贸易服务水平。调整进出口商品结构，扩大科技含量高、附加值高的机电产品和高新技术产品出口，促进对外贸易结构升级。支持更多企业取得进出口经营权，实现外贸经营主体多元化。积极参加省政府组织的各种大型对外经贸活动。鼓励有实力的企业“走出去”，努力拓展国际市场。

大力发展第三产业。加大旅游宣传推介力度，进一步整合旅游资源，加快旅游服务中心建设，完善配套设施，做大做强漂流、温泉等旅游品牌，打造一批特色旅游、生态旅游和休闲度假旅游精品景区，提升旅游业发展水平。加快发展物流业，推进一批重点物流项目建设。加快推进五洲商贸城、国际汽车城、农副产品批发市场等大型专业市场建设。大力推进“万村千乡市场工程”，推进城市连锁和超市向农村延伸，大力发展“农家店”，逐步构建新型农村市场流通网络。积极推进电子商务的发展，大力培植会展、金融、保险、信息、咨询等成长性强的现代服务业。

三、突出抓好招商引资，增强工业集群发展能力

提升园区建设水平。以园区整体规划和产业规划为先导，进一步加大以清远高新技术产业开发区、佛山（清远）产业转移园区为龙头的工业园区和产业转移园区基础设施及生活配套设施建设力度，加快园区服务平台建设，提高园区产业吸引力，促进企业向园区集中，产业向园区集聚，避免处处办厂、村村冒烟，切实维护良好生态环境。

强化招商引资工作。创新招商引资理念，从招商引资向招商选资转变。完善招商引资激励机制，加强大项目和新批外商投资企业的跟踪服务，力促新签项目早建设、早投产。扎实做好引进项目前期工作，提高项目履约率和资金到位率。切实做好为企业服务工作，促进现有企业增资扩产，做大做强。认真做好第五届泛珠三角经贸洽谈会、第五届山区经贸洽谈会、第五届中小企业博览会等各类经贸洽谈会的组织工作。

提高自主创新能力。构建以企业为主体的自主创新体系，扶持有条件的企业搞好研发，特别是扶持骨干中小企业建设省、市级工程技术研发中心。大力实施品牌带动战略，总结推广“老字号”企业创品牌、壮品牌、用品牌的典型经验，加强技术创新、品牌创新、管理创新和组织创新，争创更多的中国驰名商标、广东省著名商标和名牌产品，以增强产品的核心竞争力。

四、加强“三农”工作，促进农村发展农民增收

大力推进农村基础设施建设。积极推进城乡水利防灾减灾工程和农田水利基本建设，加快省人大议案农村机电排灌工程建设，精心组织实施标准农田建设项目。抓紧开展小流域综合整治，着力解决农村饮水安全问题，抓好佛冈县“全国农村饮水安全工程示范县”建设工作。推进林业生态县达标创建工作。

加快现代农业发展。从今年起实施现代农业“两大调整”战略。加快清远（清新）现代农业示范园区建设。进一步加快农民专业合作经济组织和农产品行业协会建设。积极培育“公司+基地+农户”模式的农业龙头企业，增强带动农民增收能力。继续引导、扶持养殖业的发展，促进种养比例进一步调整。继续推进“一乡一品”活动，积极推进名优家禽、特色农业的标准化、基地化、链条化、品牌化生产，提高农产品质量和效益。加快农业科技创新，力争在新品种培育、动物疫病和植物有害生物防控、关键农业设施和农机装备上有重大突破。加强农产品质量安全管理，建立和完善农业标准体系、农产品质量检测体系、农产品质量认证体系。大力培育、发展农业名牌产品和绿色食品、有机食品。

扎实推进社会主义新农村建设。落实中央和省各项强农惠农政策，加大财政支农力度，改善农业生产条件，进一步减轻农民负担。将新农村建设与扶贫开发、乡村管理结合起来，继续开展村企共建新农村活动，采取产业带动、合作开发、直接参与、智力帮扶、公益捐助等模式共建新农村。从环境卫生综合整治入手，加快农村饮水、道路、沼气、电力、文化、卫生等基础设施建设，争取用3年时间，基本改变农村环境卫生状况和村容村貌。

五、加强资源节约和环境保护，促进可持续发展

推进资源节约和集约利用。加快全市土地利用规划修编工作，做好全国第二次土地调查，年内完成农村土地调查工作。大力清理闲置土地，推进旧村、旧区、旧厂整合，提高现有土地利用效益。优化土地布局和结构，确保重点项目和产业建设用地需求。规范矿产资源开发和管理。在全社会倡导节约意识，调动全民支持和参与资源节约型、环境友好型社会建设的积极性。

着力抓好环境保护和生态建设。落实节能减排工作责任制，今年起对各县（市、区）及全市百家重点耗能企业实行绩效考核。着力抓好火电、冶金、化工、建材等重点行业和重点企业的节能减排。积极推广节约、替代、循环利用和治理污染的先进适用技术，大力推进清洁生产。继续抓好清城区国家级循环经济标准化试点工作。严格执行环境管理各项制度，建立固定资产投资项目节能、环保评估和审查制度。加快市污染源监控中心建设步伐，提高环境应急和污染物总量减排监测能力。严厉查处露天焚烧废旧物资行为，加大小作坊式拆解固体废物污染环境行为整治力度。加强监管，巩固大燕河、乐排河流域整治成果。加快高新区及各县（市、区）污水处理厂建设。加强饮用水水源地保护。加快推进“市肺”工程建设。全面开展污染源普查。认真开展环保执法专项行动，切实解决关系群众切身利益的突出环境污染问题。

六、强化基础设施建设，优化经济发展环境

着力抓好重点项目建设。继续抓好2007年未完工重点项目建设，着力推进2008年计划安排的117个重点项目建设，尤其要加快清远新火车站配套工程以及飞来湖防灾减灾综合工程等列入省重点工程建设项目的推进。围绕结构调整和发展方式转变，谋划一批大型项目，加强重大项目储备。

加强电源电网建设。加快完成清远抽水蓄能电站、阳山燃煤发电厂“上大压小”工程、侨远电厂等重大电源建设项目的前期工作，促其尽快开工建设。加快电网建设，确保完成500千伏贤令山、220千伏燕河等输变电工程建设，增强电网防灾抗灾能力。

加快交通基础设施建设。完善高速公路网和中心区域综合交通规划。确保完成清连公路高速化改造、清远城西大道、石龙和德龙产业大道、国道323线连山吉田至鹰扬关段、省道261线连南三江至水足塘段等工程建设。加快推进清远大桥抢修加固、省道253线银英公路英德段、国道106线佛冈段、国道323线连州至连南段等工程建设，切实做好广（州）乐（昌）、佛（山）清（远）从（化）、二（连浩特）广（州）、汕（头）湛（江）等高速公路清远段的前期工作，争取尽快立项动工。加快农村公路建设，力争完成1200公里农村公路路面硬化任务。积极发展农村公共交通，加快完善全市农村客运班线。大力推进北江中游航道整治和连江渠化加固维修工程建设，配合做好英德西牛航道枢纽工程建设，逐步完善综合运输体系。

提升城市规划、建设与管理水平。按照建设“绿色经济强市、岭南宜居名城、华南休闲之都”的定位，进一步完善城乡专项规划和详细规划。配合国家、省做好主体功能区规划编制的各项前期工作。启动“湖城”建设分步实施计划，着力推进清远水利枢纽工程建设，加快飞来湖建设步伐，把市中心区逐步建成具有清远特色的“湖城”、“水城”。加快城市主干路网、公园绿地和公共配套设施建设。加快市垃圾焚烧发电厂和垃圾填埋场二期工程、供电、供水、燃气、雨水污水排放管网建设与改造。推进各种人文设施建设，完善城市服务功能。规范房地产市场行为，促进房地产业健康发展。加快县（市）城区和中心镇建设步伐，不断提高城镇化水平。大力推进城中村和旧城改造，完成一批道路、小区、景观带（区）的绿化、亮化、美化工程建设。启动“城乡保洁工程”，加大城乡环境卫生整治力度，集中力量查处违法违章建筑和整治城市“六乱”现象，提高城市文明程度。

七、加强财税金融工作，增强发展保障能力

加强财税征管。坚持依法治税，规范非税收入管理，积极培育税源，促进财政增收。加强国有资产和资源经营收益管理。加快推进各项公共财政改革，进一步完善公共财政体系。继续拓宽国库集中支付范围，推进县级国库制度改革。加强财政性资金的绩效评价工作，提高财政性资金使用效益。加大专项资金和重大投资项目的审计监管力度，继续完善“收支两条线”管理。调整优化财政支出结构，严格控制一般性支出和行政事业经费的增长，集中财力确保重点项目支出，提高对教育、医疗、社保等民生领域的保障能力。

优化金融环境。继续做好停业整顿城市信用社退出市场工作。鼓励发展农村新型金融组织，加大金融对中小企业和“三农”支持力度。鼓励金融产品创新，提高金融服务水平，增强金融企业综合竞争力。加强和改进金融监管，提高信贷比例，防范金融风险。

培育资本市场。积极培育优质企业上市后备资源，争取一批企业入围省上市后备企业。鼓励、扶持企业股份改造与改制上市，稳步推进资本市场发展。

八、注重改善民生，共建共享改革发展成果

完善城乡统筹就业机制。加强劳动力市场建设，进一步完善创业、就业和培训服务体系。统筹做好新增劳动力就业、下岗失业人员再就业和农村富余劳动力转移工作，新增就业岗位8.5万个，培训富余劳动力4.2万人次。全面落实促进就业再就业扶持政策，重点做好下岗失业人员、大中专毕业生、退伍转业军人、农村贫困家庭等群体的培训与就业工作。宣传贯彻好新《劳动合同法》，健全劳动关系调整机制，确保劳动关系和谐稳定。

完善社会保障体系。以非公有制经济组织人员、城镇灵活就业人员和农民工为扩面重点，不断扩大社会保险覆盖面。严格社保基金管理，确保基金安全。全面启动被征地农民养老保险和城镇居民医疗保险，完善农民工和国有、集体困难企业职工医疗保险制度。扩大城镇职工基本养老保险和基本医疗保险覆盖面。

完善社会救助体系。加强灾区受灾群众救济工作，确保受灾群众基本生活得到保障。抓好以城乡低保为重点的社会救助工作，把农村人均收入低于1500元的困难群众全部纳入低保救济，做到动态管理下的应保尽保。做好“五保”和残疾人等弱势群体的救助工作，加快市救助站、福利院等民心工程建设。加强法律援助和司法救助，保障困难群众合法权益。启动廉租房、经济适用房建设，努力解决城镇低收入家庭住房困难问题。

九、积极发展社会各项事业，大力推进和谐清远建设

强化教育基础和优先发展战略地位。继续落实免费义务教育制度和教育救助政策，进一步完善农村、城镇义务教育经费保障机制，从春季学期起将城镇义务教育阶段学生纳入免学杂费和课本费范围。保障家庭经济困难学生接受教育。加强幼教工作，切实解决适龄幼儿入园入托问题。鼓励社会各界捐资办学，落实办学优惠政策，促进民办教育健康发展。继续依法落实财政投入政策，加快发展普及高中阶段教育，不断提高高中阶段毛入学率，切实加强师资队伍建设，不断提高教育水平；推进义务教育规范化学校建设，继续开展教育创强工作。加强职业技能教育，完成市高级技工学校“二次创业”工程建设，完善市职业技术学院的内涵建设，扎实做好迎评促建工作。

加快卫生事业发展。进一步完善公共卫生体系建设，提高公共卫生服务水平，增强应对突发公共卫生事件的能力。加强镇级卫生院建设。不断扩大新型农村合作医疗覆盖面，确保农村新型合作医疗参保率稳定在97%以上。加快建立城市社区卫生服务体系，以街道办事处为单位普及卫生服务中心，为居民提供基本卫生服务。规范医疗服务行为，保障医疗质量和医疗安全。完善卫生投入长效机制。完成市人民医院新院区建设，加快市中医院建设，做好市妇幼保健院建设的各项前期工作。深入开展爱国卫生运动。

大力发展文化、科技、体育事业。积极稳妥推进文化体制改革。加大文化遗产保护力度。继续组织开展“送戏下乡、送书下乡、送影下乡”活动。进一步推进广播电视数字化工程，完成20户以上已通电而未通广播电视自然村的“村村通”广播电视工程检查验收工作。加强城乡公益性文化、体育设施建设，不断完善公共文化服务体系。继续举办好广场文化艺术节等各类节庆文化活动。加强产学研合作开发，大力推进科技创新专业镇建设。加大知识产权保护力度。扎实推进信息化建设。继续开展以“全民健身与奥运同行”为主题的群众体育活动，抓好“农民健身工程”工作。承办好国际漂流大赛和市龙舟赛。加强思想道德、职业道德教育，加大群众性精神文明创建力度，做好全国文明城市申报工作。

强化安全生产工作。进一步落实安全生产责任制，强化安全生产管理和监督，遏制重大事故，杜绝特大事故。巩固产品质量和食品安全专项整治成果，强化对产品和食品生产、经营、销售等环节的监管。组织开展食品安全示范县、镇活动。建立健全长效机制，确保产品质量和食品安全。

切实抓好物价稳定工作。抓好市场供给，千方百计确保粮食、肉类和食用油等重要商品的供给，保障主要农产品基本供应不脱销、不断档。严格控制涨价项目出台，加强价格监控，依法打击串通涨价、囤积居奇、哄抬物价等违法违规行为，努力保持全市物价水平的基本稳定。

完善社会管理机制。加快建立政府应急体系，及时应对和妥善处置各类突发事件。深入开展打击制假售假、商业欺诈、走私贩私、传销等专项整治，维护市场秩序。加快推进社会信用体系建设。继续加强信访工作，强化群体性事件预防处置机制，加大矛盾纠纷排查调处力度，有效预防和化解各种社会矛盾。继续妥善解决革命老区和移民历史遗留问题。突出以防为主，不断完善社会治安防范体系，深入开展平安社区建设，构建严密的社会治安防护网。坚持严打方针不动摇，严厉打击各种黑恶势力、严重刑事犯罪和“两抢”、“两盗”等多发性犯罪。进一步增强社区警务力量，把社会治安综合治理各项措施落实到基层，为科学发展创造和谐稳定的环境。

大力发展社会各项事业。加大统筹解决人口问题的力度，深入开展“两无”活动，争取“两无”达到全省平均水平；综合治理出生人口性别比偏高问题，切实加强流动人口管理和服务，继续稳定低生育水平。进一步加强全民国防教育，做好“双拥模范城”复评工作，加强民兵预备役工作，促进军政军民团结。加强少数民族工作，大力推进民族团结，提高依法管理宗教事务能力。做好第二次经济普查工作。扎实做好妇女儿童工作。继续做好统计、外事、侨务、对台、移民、红十字、人防、气象、地震、档案、方志、殡改等各项工作。

十、加强政府自身建设，提高推动科学发展能力

全面加强政府自身建设，努力建设法治、高效、廉洁和服务型政府，提高驾驭全局、管理和服务社会的能力。

深化改革。转变政府职能，进一步强化社会管理和公共服务职能。深入推进行政审批制度改革。深化行政管理体制改革，完善公务员制度和机关事业单位工资收入分配制度，稳妥推进事业单位制度改革和乡镇机构改革。改革和完善领导干部政绩考核评价体系。全面推进华侨农场改革与发展，促进和谐侨区建设。继续加大教育、文化、卫生等资源整合力度，优化公共服务。深化国有企业改革，推进国有企业保值增值。深化投融资体制改革。深化医疗卫生和药品采购体制改革。

严格依法行政。继续开展“五五”普法教育，贯彻落实行政许可法和全面推进依法行政实施纲要，建立健全权责明确、行为规范、监督有效、保障有力的行政执法体制。推行行政执法评议考核制、行政执法过错责任追究制等各项行政执法责任制，规范行政执法，强化执法责任。继续加强和完善行政服务中心和行政审批电子监察系统建设，全面落实“一站式”办公、首问承诺制等工作制度。加强民主政治建设，认真落实人大及其常委会的各项决议决定，坚持向人大及其常委会报告工作，自觉接受监督。主动加强与人民政协的联系并向其通报工作，积极支持政协委员发挥参政议政作用。及时、高质办理人大议案建议和政协委员提案。进一步加强与各民主党派、工商联、无党派人士的联系与沟通，广泛听取工会、共青团、妇联等人民团体的意见。完善公众参与、专家论证和政府决策相结合的行政决策机制，对涉及全市经济社会发展大局、关系人民群众切身利益的重大事项，实行听证、专家咨询制度。认真受理来自社会各界的意见和建议，自觉接受新闻媒体、人民群众和社会各界的监督，推进政府行政决策的民主化、科学化和制度化，努力提高政府的行政力和公信力。

强化廉洁从政。全面贯彻落实党风廉政建设各项规定，进一步完善教育、制度、监督并重的惩治和预防腐败体系。加强对权力的监督，特别是对领导干部、人财物管理使用的监督和制约。坚持推进改革，创新机制，注重从源头上遏制腐败。深入开展执法监察、廉政监察和效能监察，健全经济责任审计制度，坚决查处违法违纪案件。全体公务员都必须牢记使命，解放思想，真抓实干，依法行政，做到一心一意谋发展，聚精会神搞建设，千方百计保民生，不断优化我市的政务环境。

同志们，我们已站在新的发展起点上，历史赋予我们的责任更重大，使命更光荣。让我们更加紧密地团结在以胡锦涛同志为总书记的党中央周围，在市委的正确领导下，高举中国特色社会主义伟大旗帜，坚持以科学发展观统领经济社会发展全局，进一步解放思想，开拓创新，扎实工作，为推动经济社会又好又快发展、争当山区科学发展排头兵而努力奋斗！
